# Long Non-Coding RNA GAPLINC Promotes Tumor-Like Biologic Behaviors of Fibroblast-Like Synoviocytes as MicroRNA Sponging in Rheumatoid Arthritis Patients

**DOI:** 10.3389/fimmu.2018.00702

**Published:** 2018-04-10

**Authors:** Bi Yao Mo, Xing Hua Guo, Meng Ru Yang, Fang Liu, Xuan Bi, Yan Liu, Lin Kai Fang, Xi Qing Luo, Julie Wang, Joseph A. Bellanti, Yun Feng Pan, Song Guo Zheng

**Affiliations:** ^1^Department of Internal Medicine, Division of Rheumatology, The Third Affiliated Hospital of Sun Yat-sen University, Guangzhou, China; ^2^Department of Internal Medicine, Division of Rheumatology, The Affiliated Hospital of Shenzhen University, Shenzhen, China; ^3^Center for Clinic Immunology, The Third Affiliated Hospital of Sun Yat-sen University, Guangzhou, China; ^4^Department of Medicine, Division of Rheumatology, Hershey Medical Center at Penn State University, Hershey, PA, United States; ^5^Department of Pediatrics and Microbiology-Immunology, Georgetown University Medical Center, Washington, DC, United States

**Keywords:** rheumatoid arthritis, fibroblast-like synoviocytes, long non-coding RNAs, LncRNA GAPLINC, cell behaviors regulation

## Abstract

Rapidly accumulating evidence has now suggested that the long non-coding RNAs (LncRNAs), a large and diverse class of non-coding transcribed RNA molecules with diverse functional roles and mechanisms, play a major role in the pathogenesis of many human inflammatory diseases. Although some LncRNAs are overexpressed in plasma, T cell, and synovial tissues of patients with rheumatoid arthritis (RA), there is a dearth of knowledge in what role these transcripts play in fibroblast-like synoviocytes (FLSs) of these patients. Here, our studies showed that GAPLINC, a newly identified functional LncRNA in oncology, displayed a greater degree of expression in FLSs from RA than in patients with traumatic injury. GAPLINC suppression in RA-FLS cells revealed significant alterations in cell proliferation, invasion, migration, and proinflammatory cytokines production. Additionally, we performed a preliminary bioinformatics analysis of GAPLINC gene sequence in order to find its target molecules, using miRanda, PITA, RNAhybrid algorithms, Kyoto encyclopedia of genes and genomes, and gene ontology analysis. Since the results predicted that some of microRNAs and mRNA may interact with GAPLINC, we simulated a gene co-action network model based on a competitive endogenous RNA theory. Further verification of this model demonstrated that silencing of GAPLINC increased miR-382-5p and miR-575 expression. The results of this study suggest that GAPLINC may function as a novel microRNAs sponging agent affecting the biological characteristics of RA-FLSs. Additionally, GAPLINC may also promote RA-FLS tumor-like behaviors in a miR-382-5p-dependent and miR-575-dependent manner. Based upon these findings, LncRNA GAPLINC may provide a novel valuable therapeutic target for RA patients.

## Introduction

Rheumatoid arthritis (RA) is one of the most chronic debilitating inflammatory autoimmune diseases characterized by synovitis and progressive destruction of joint cartilage and bone damage (1, 2). The disease is not only a major source of patient disability but also carries with it a substantial burden of illness for both the individual and society. Several cell types, including T cells, B cells, macrophages, fibroblast-like synoviocytes (FLSs), and the complex interaction of many pro-inflammatory cytokines play a major role in the pathogenesis of this disease (3). Among these, the FLSs are the main effector cells responsible for initiating and driving both the inflammatory process as well as the invasive nature of rheumatoid synovium (4–7). A congeries of evidence now suggests that the RA-FLSs, when activated in a chronic inflammatory environment, display many features of tumor cells. In due course, these cells eventually escape the growth limitations of contact inhibition, enhance migration, and acquire invasive ability, features essential in the pathogenesis of RA (8, 9).

Rapidly accumulating evidence now suggests that long non-coding RNAs (LncRNAs), known to play important roles in various biological processes, may also be critically involved in the pathogenesis of many autoimmune diseases including RA (10, 11). Although a number of LncRNAs have been found, knowledge of their function and physiological/pathological significance is still in its infancy. GAPLINC, a novel long non-coding RNA, positive CD44 Regulator, was previously described in gastric cancer and colorectal cancer (CRC) tissues. Upregulation of GAPILNC has been observed in these carcinomas and predicts an unfavorable prognosis. GAPLINC has been reported to markedly promote migration, invasive behavior, proliferation and metastasis of tumor cells, indicating that this LncRNA may be an oncogene in cancers (12–14). Recent several studies have shown that RA FLSs display many features of tumor cells when activated in a chronic inflammatory environment. Based upon these observations of GAPLINC on tumor behaviors, the present study was performed to determine whether GAPLINC may influence the pathological phenotypes of RA-FLS.

In this report, we compared the expression of GAPLINC in FLSs obtained from patients with RA with common traumatic injury. Moreover, after suppression of GAPLINC by RNA interference technology, functional assays were used to detect proliferation, migration, and invasion of RA-FLSs. We also performed a preliminary bioinformatics analysis of GAPLINC gene sequence to identify its target molecules using acknowledged databases [miRanda, PITA, RNAhybrid, Kyoto encyclopedia of genes and genomes (KEGG)]. These data were used to simulate a gene co-action network of GAPLINC based on a competitive endogenous RNA (ceRNA) theory. In addition, we verified some of candidate microRNAs and identified the silencing effects of GAPLINC upregulation on the expression of miR-382-5p and miR-575. Therefore, we postulate that a functional long non-coding RNA, GAPLINC, affects the biological characteristics of RA-FLSs, and all these may be due to a mechanism of microRNAs sponging.

## Materials and Methods

### Patients

All synovial specimens were taken from patients who underwent knee replacement surgery, knee synovial debridement or meniscal repair surgery at the Third Affiliated Hospital of Sun Yat-sen University, from January 2015 to July 2017. Subjects included 11 patients with RA (consistent with the criteria of the American College of Rheumatology) and 3 patients with severe joint trauma who had no other joint abnormalities or systemic disease. All samples were obtained from discarded tissues. The research was approved by the ethics committee of the Third Affiliated Hospital at the Sun Yat-sen University (No.[2017]2-169) and all subjects were given the written informed consent in accordance with the Declaration of Helsinki.

### Synovial Cell Culture and Cell Characterization

Synovial tissues were dissected free of fat, blood vessels, and fibrous tissue, rinsed 2–3 times with PBS buffer, shredded into fragments of approximately 1 mm^3^, transferred to culture flasks, and tiled individually with intervening space of about 5 mm. The flasks contained an appropriate amount of DMEM (Gibco, USA) culture medium supplemented with 10% fetal bovine serum (FBS, Gibco, USA) and were placed upright for tissue adherence in a 37°C, 5% CO_2_ thermostatic incubator. After 4 h, the culture flasks were carefully laid flat and cell cultures were continued. Generally, FLSs migrated out from the tissue explant on the second or third day and were grown to approximately 95% confluency within 3 weeks. Fresh culture medium was changed every 2–3 days. The adherent cell monolayer (coverage > 80%) was removed by a trypsin–EDTA solution, collected, re-suspended, and implanted as an adherent cell subculture. The fibroblast-like synovial cells from passages 3 to 6 were used for the following experiments.

The morphology of FLSs was observed under the light microscope. The surface markers (CD55, CD90, CD44, CD14, CD68) of FLS at passages 3 were detected by flow cytometry. Commercial monoclonal antibodies CD44FITC, CD14FITC, CD90FITC, CD68FITC, CD55PE (Biolegend, USA) were used for characterization, and each experiment included cell preparations treated with isotype-matched control antibodies as methodologic controls. The flow cytometric analysis was performed on a BD FACS Calibur (BD Biosciences, USA) and data were collected using BD CellQuest software (BD Biosciences, USA).

### Transfection and RNA Interference

The chemically synthesized small interference RNAs (siRNAs) used in this study were purchased from GenePharma (Shanghai, China). RA-FLSs were seeded in 6-well plates (1 × 10^5^ cells/well) or in 96-well plates (2.0 × 10^3^ cells/well) 24 h prior to transfection according to different needs. When RA-FLSs achieved 60–70% confluency, siRNAs were transfected using Lipofectamine^®^ RNAiMAX (Invitrogen, USA) at a final concentration of 50 nM according to the manufacturer’s instructions. The supernatants of transfected RA-FLSs were replaced after 24 h with fresh culture medium and incubated for an additional 24–96 h based upon experimental need. The inhibition efficiency of specific siRNAs was detected by qRCR. The transfected RA-FLSs were then used in individual experiments described below.

### Quantitative Real-Time Reverse Transcription PCR (qRT-PCR)

Total cellular RNA was extracted using the RNAiso Plus reagent (Takara, Japan). For detecting the expression of LncRNA GAPLINC, equal amounts of RNA from different samples were reverse-transcribed using the PrimeScript^®^ RT reagent Kit with gDNA Eraser (Takara, Japan) and underwent PCR with SYBR^®^Premix Ex TaqTMII kit (Takara, Japan) on the ABI 7500 Fast real-time PCR amplification equipment. Sequence of primer sets and specific siRNA sequences used in this study are listed (Table S1 in Supplementary Material). For detecting the expression of microRNAs, the extracted RNA was reverse-transcribed and underwent PCR reaction using special microRNA detection kit (GenePharma, China). The specific primers of microRNAs and U6 were contained in the kits. Ct values of each sample were analyzed and 2^−ΔΔCt^ was calculated to indicate relative quantification of target genes to control gene GAPDH/U6.

### Cell Viability Analysis by CCK-8

Cell proliferation was measured using a cell counting kit-8 (Dojindo Laboratories, Japan) assay according to the manufacturer’s protocol. Initially, approximately 2.0 × 10^3^ RA-FLSs in a volume of 100 µl DMEM with 10% FBS were planted in each well of 96-well plate. Cells in logarithmic growth phase were then transfected 24 h following the above method. Supernatants of transfected cells were replaced with fresh culture medium and continued to be incubated for another 24, 48, 72, and 96 h. At each experiment point, 10 µl of the CCK-8 solution were added to each well and incubated at 37°C for 1.5 h. Absorbance at 450 nm was measured to evaluate cell viability using a Microplate Reader (Thermo Scientific, USA).

### Cell Migration Assay

Transwell chambers with a pore size of 8-µm (Corning, USA) were used for cell migration and invasion assays. Approximately 8 × 10^3^ cells transfected for 48 h were resuspended in serum-free medium and seeded in the upper chamber following which an additional 600 µl culture medium containing 10% FBS were added into the lower chamber as chemoattractant. After 12 h incubation at 37°C, the cells that had migrated through the membrane were fixed with 4% paraformaldehyde (Boster, China) for 20 min and stained with crystal violet for another 20 min, followed by microscopic visualization and counting. The mean number of migrated cells was recorded in five randomly selected fields at 50× magnification in every membrane.

### Cell Invasion Assay

Cell invasion was determined using the same transwell chamber methodology described previously. The upper chamber wells were coated individually with 30 µl diluted matrigel (BD Biosciences, USA). The matrigel was diluted 1:10 with DMEM. After air-drying and concretion of matrigel, approximately 1 × 10^4^ cells transfected for 48 h were resuspended in serum-free DMEM and seeded into the upper chamber and the lower chamber was covered by 600 µl culture medium containing 10% FBS. After 24 h of culture at 37°C, subsequent procedures were conducted as described with the migration assays.

### Assessment of Multiple Excreted Factors by ELISA

Approximately 1.0 × 10^5^ RA-FLS cells were cultivated in each well of six-well culture plates to obtain high confluency (>80%). The cell supernatants were collected in 48 h after transfection and analyzed for proinflammatory cytokines [interleukin-6 (IL-6), IL-8] and matrix metalloproteinases-9 (MMP-9) with ELISA kits (R&D Systems, USA) following the instructions.

### Bioinformatics Analysis

Genbank is a public functional genomics database supporting data submissions. The GAPLINC gene sequences were downloaded from NCBI Genbank (Gene ID: 100505592). The target microRNAs of GAPLINC were predicted using miRanda, PITA, and RNAhybrid algorithms. LncRNA-microRNA seed region, microRNA recognition elements, LncRNA-microRNA binding-free energy were the relevant elements taken into consideration for possible interactions. Those microRNAs, which have the same or similar seed sequence of GAPLINC as well as better comprehensive assessment were selected as candidates. The microRNAs intersections of three databases are presented by Venn graph. Target genes of selected microRNAs were predicted using TargetScan, miRDB, and miRanda software as well as high throughput CLIP-seq data. The gene set described above was obtained by biological pathway enrichment analysis based on KEGG database and gene ontology (GO) analyses. The outcome was used to construct of an LncRNA GAPLINC-microRNA-mRNA prediction model based on ceRNA hypothesis.

### Statistics

Statistical analysis was performed by SPSS version 20.0 software. The experimental data were presented as mean ± SD. Multiple groups of samples were analyzed by one-way ANOVA, pairwise comparisons were adjusted by Bonferroni method and differences were considered statistically significant when *P* < 0.05.

## Results

### Overexpression of LncRNA GAPLINC in RA-FLSs

**Figure 1:**
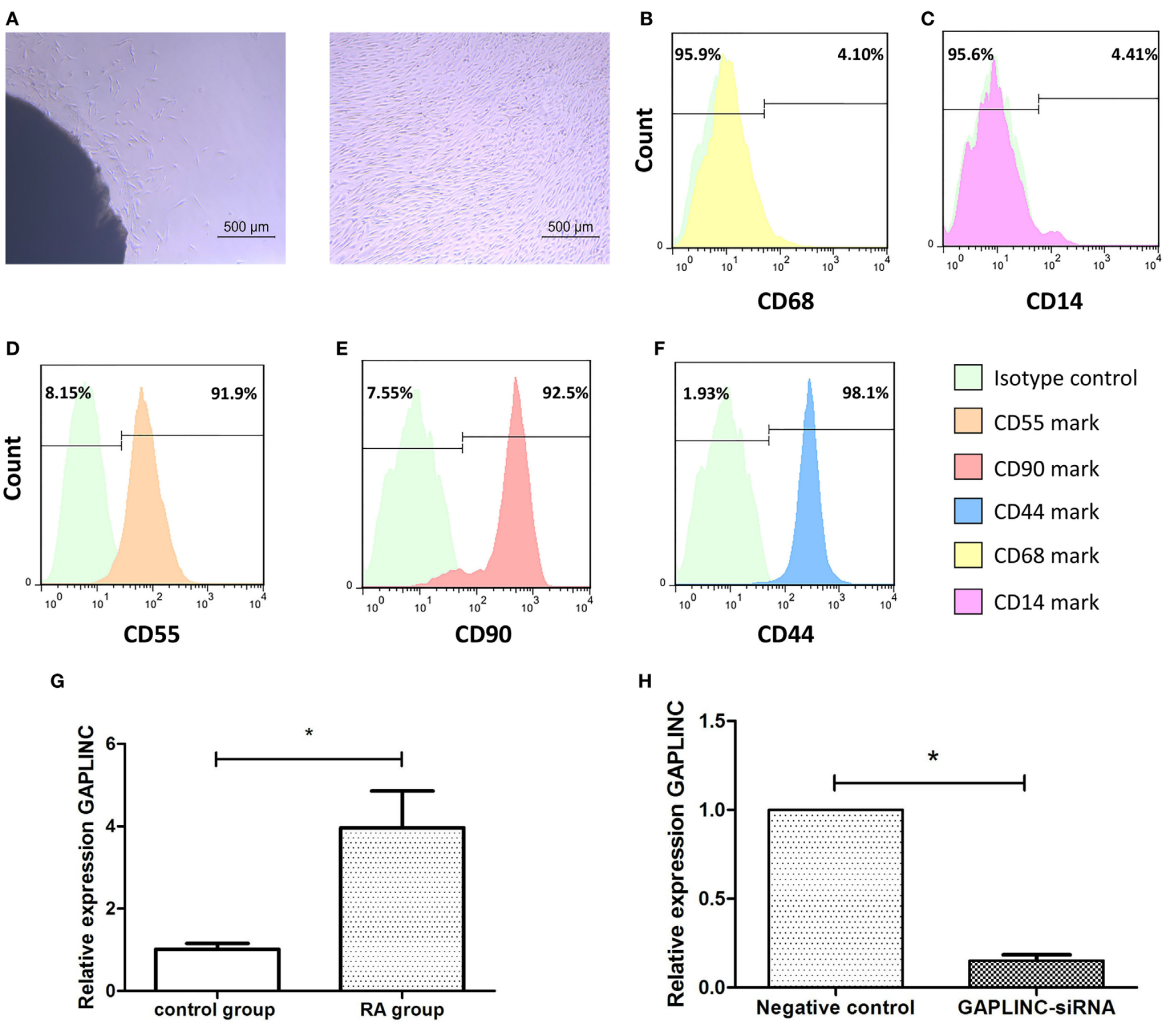
The cell characterization and the relative expression of LncRNA GAPLINC in different groups. **(A)** The morphology of rheumatoid arthritis (RA)-fibroblast-like synoviocytes (FLSs) were observed under the light microscope. The left graph showed primary fibroblast-like synoviocytes of RA crawled out from synovial tissues: the right graph showed the RA-FLSs at passages 3 have unique morphology. In **(B–F)**, the surface markers (CD55, CD90, CD44, CD14, CD68) of RA-FLS at passages 3 was detected by Flow cytometry. The left was Isotype control map; the right was surface mark map. Cells were identified by CD68 **(B)** and CD14 **(C)** negative staining, coupled with positive staining for CD55 **(D)**, CD90 **(E)**, and CD44 **(F)** staining. The diagrams revealed CD55, CD90 and CD44 expression rate of RA-FLS cell at passage 3 highly up to 90%. **(G)** LncRNA GAPLINC expression is increased in FLSs from RA patients than trauma groups analyzed by quantitative real-time reverse transcription PCR (qRT-PCR) (*P < 0.05). **(H)** GAPLINC-small interference RNA efficiently interferes with GAPLINC expression in RA-FLSs detected by (qRT-PCR) (*P < 0.05).

Fibroblast-like synoviocytes isolated from RA patients (RA-FLS) with unique spindle morphology were cultured and observed *in vitro* (Figure 1A). Characterization of FLS could include positive staining of surface markers for VCAM-1, CD44, CD55, CD90, and cadherin-11, coupled with negative staining for macrophage markers such as CD14 or CD68 (15). Subsequently, the expression of surface markers (CD55, CD90, CD44, CD14, CD68) of FLS by flow cytometry was chosen for cell characterization. The graphs identified positive staining cells for CD55, CD90, CD44, coupled with CD14 and CD68 negative staining. The expression of surface markers (CD55, CD90, CD44) of FLS at passage 3 was all highly up to 90% (Figures 1B–F).

The use of qRT-PCR was a second to distinguish molecular differences of the FLSs of RA patients from those of trauma patients. The relative mRNA expression of GAPLINC was higher in RA-FLSs compared with trauma-FLSs and this difference was statistically significant (*P* < 0.05) (Figure 1G). We next sought to investigate whether the elevated expression of GAPLINC could be regulated by specific siRNA in RA-FLSs. The GAPLINC targeted RNA interference, GAPLINC-siRNA-461, was successfully constructed from GenePharma (Shanghai, China) and the inhibition efficiency was detected by qRT-PCR. The results showed that compared to the negative control (NC-siRNA) group, GAPINC silencing by siRNA-461 for 24 h led to significant decrease of mRNA expression by (84.85 ± 3.32)% (*P* < 0.05) (Figure 1H).

### GAPLINC Knockdown Inhibits RA-FLSs Proliferation

**Figure 2:**
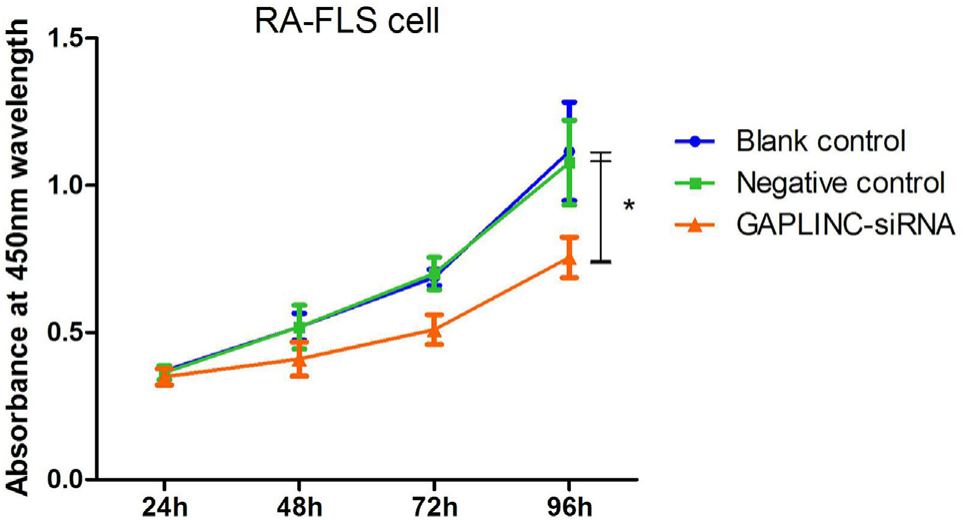
Knocking-down GAPLINC in rheumatoid arthritis-fibroblast-like synoviocytes decreases cell proliferation. Cell growth was measured using a cell counting kit-8 assay. The absorbance at 450 nm wavelength of GAPLINC-small interference RNA group were significantly reduced at 24, 48, and 72 h, largely maintained at 96 h compared to two control groups, *P < 0.05.

To determine whether the elevated LncRNA GAPLINC expression on RA-FLSs was functional, we conducted GAPLINC-siRNA silencing to explore whether this knockdown altered the proliferation of RA-FLSs using the CCK8 assay. Growth curves of RA-FLSs showed proliferation levels of these cells at different time points (Figure 2). The absorbance at 450 nm wavelength of NC group at four time points was close to the blank control group. In the GAPLINC-siRNA group, an inhibition rate in growth was first observed (15.29 ± 0.38%) at 24 h after transfection, then a significant suppression was observed (28.75 ± 2.34%) at 48 h, more apparent (36.63 ± 7.93%) at 72 h, and largely maintained (35.97 ± 3.67%) at 96 h after siRNA treatment, compared to the NC group. The data of GAPLINC-siRNA group were significantly different from the two control groups, respectively (*P* < 0.05).

### GAPLINC Knockdown Decreases RA-FLSs Migration and Invasion *In Vitro*

**Figure 3:**
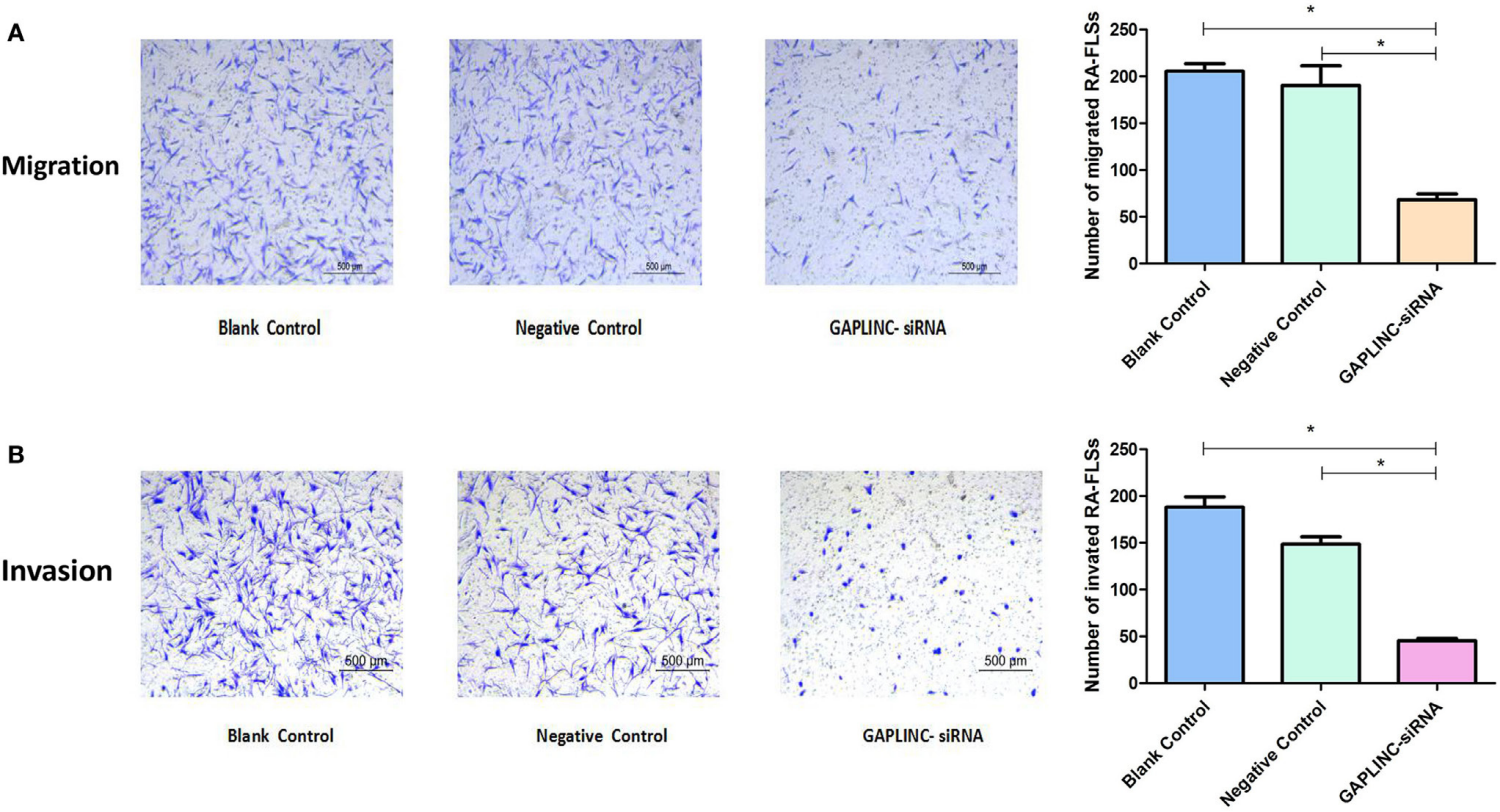
Knocking-down GAPLINC in rheumatoid arthritis (RA)-fibroblast-like synoviocytes impaired cell migration and invasion ability. The migration and invasion in RA-FLS were examined, respectively, by Transwell assay and Matrigel Transwell assay. In both **(A,B)**, left panels show representative images of transmembrane cells; right panels present quantification (means ± SD) of different groups, *P < 0.05.

Given that migration and invasion are other important features of RA-FLSs, we next examine whether GAPLINC was required for these cellular properties. In the migration assay, the transmembrane cell numbers decreased significantly in the treatment group with GAPLINC knockdown (68.0 ± 6.0) compared to values observed in the NC-siRNA group (191.0 ± 21.0) and blank control groups (206.0 ± 8.0) at 12 h (Figure 3A). At the same time, Ttranswell chambers with Matrigel were employed to detect the invasive nature of RA-FLSs at 24 h after transfection. The results showed a substantial decrease in the number of cells that penetrated the porous filter, suggesting impaired invasive ability of cells with GAPLINC suppression. Statistical analysis revealed that the numbers of membrane-invading RA-FLSs were (45.0 ± 3.0) in the GAPLINC-siRNA group compared to values in the NC-siRNA (149.0 ± 7.0) and in the blank control groups (188.0 ± 11.0), respectively (Figure 3B). These data comparisons were all statistically significant (*P* < *0.05*). The results of these assays demonstrated that LncRNA GAPLINC positively regulated RA-FLS migration and invasion.

### GAPLINC Knockdown Decreases IL-6, IL-8, and MMP-9 Production of RA-FLSs

Besides the tumor-like behaviors, RA-FLS cells also contribute to pathological destructions through expression of a variety of proinflammatory cytokines or proteinases such as IL-6, interleukin-8 (IL-8), and MMPs. The ELISA was used to assess the secretion levels of multiple factors in the supernatants of RA-FLS cells 48 h after transfection. The results revealed that GAPLINC knock-down significantly reduced IL-6 (*P* < 0.01), IL-8 (*P* < 0.05), and MMP-9 (*P* < 0.05) production of RA-FLSs when compared to the NC-siRNA control group (Figure S1 in Supplementary Material).

### Construction of a LncRNA GAPLINC-microRNA-mRNA Network Model Based on the Bioinformatics Analysis

**Figure 4:**
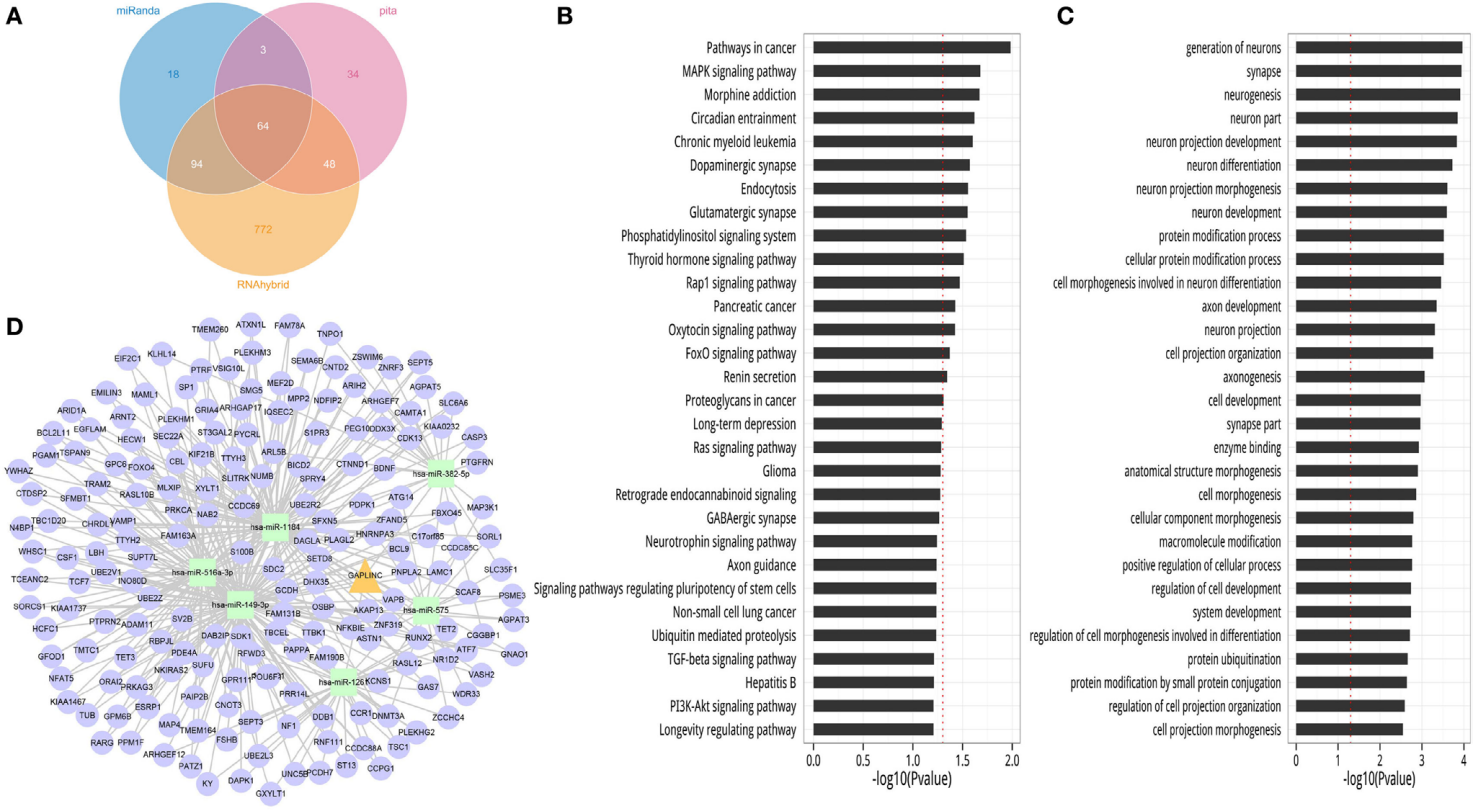
The bioinformatics analysis result of GAPLINC sequence. **(A)** The Venn graph showed 64 candidate microRNAs of GAPLINC in the intersections of miRanda, PITA, and RNAhybrid algorithms. **(B)** The classification map of KEGG analysis predicted the possible GAPLINC-related pathways. The *x*-axis is the −log10 of the *p*-value, and *p* < 0.05 was considered statistically significant. **(C)** The distribution map of gene ontology (GO) analysis revealed the possible function of GAPLINC-related genes. The *x*-axis is the −log10 of the *p*-value, and *p* < 0.05 was considered statistically significant. **(D)** The view of ceRNA module network of GAPLINC based on front six candidate microRNAs (hsa-miR-575, hsa-miR-149-3p, hsa-miR-382-5p, hsa-miR-516a-3p, hsa-miR-1184, hsa-miR-1261).

Although the results of functional assays described above suggested that LncRNA GAPLINC may affect some important characteristics of RA-FLSs, the underlying mechanism remained unclear. In order to probe the GAPLINC-associated pathway more rigorously, we performed a bioinformatics analysis using GAPLINC gene sequence and acknowledged databases. Initially, we sought related microRNAs that could interact with GAPLINC using miRanda, PITA, and RNAhybrid algorithms based on their recognition elements. The microRNA intersections in three softwares suggested that 64 microRNAs (e.g., hsa-miR-575, hsa-miR-149-3p, hsa-miR-382-5p, hsa-miR-516a-3p, hsa-miR-1184, hsa-miR-1261, hsa-miR-3127-5p, hsa-miR-3127-5p, hsa-miR-4649-3p) could be highly related to GAPLINC, and therefore, could be selected as candidates (shown by Venn graph, Figure 4A). Based upon these considerations, TargetScan, miRDB, and miRanda databases as well as high throughput CLIP-seq data were selected to explore the target genes of those related microRNAs. The results showed that 9559 target genes were involved. Subsequently, the genes described above were employed in biological pathway enrichment analysis assays based on the KEGG database and Gene ontology (GO) enrichment analysis. The GAPLINC-related pathways using KEGG database prediction demonstrated involvement of a part of the signaling pathways including MAPK signaling pathway, Rap1 signaling pathway, Ras signaling pathway, PI3K-Akt signaling pathway (Figure 4B). Another distribution map of GO analysis revealed the possible function of GAPLINC-related genes (Figure 4C). These data were of great assistance in the selection of suitable target molecules of GAPLINC and as a result certain superior candidates were selected to construct a possible LncRNA-microRNA-mRNA network model (Figure 4D). In this investigation, we were particularly interested in three candidate microRNAs (hsa-miR-575, hsa-miR-149-3p, hsa-miR-382-5p), all of which were characterized with high comprehensive databases scores. It was decided to first verify their properties in the following sets of experiments.

### GAPLINC Knockdown Upregulates miR-382-5p and miR-575 Expression in RA-FLSs

**Figure 5:**
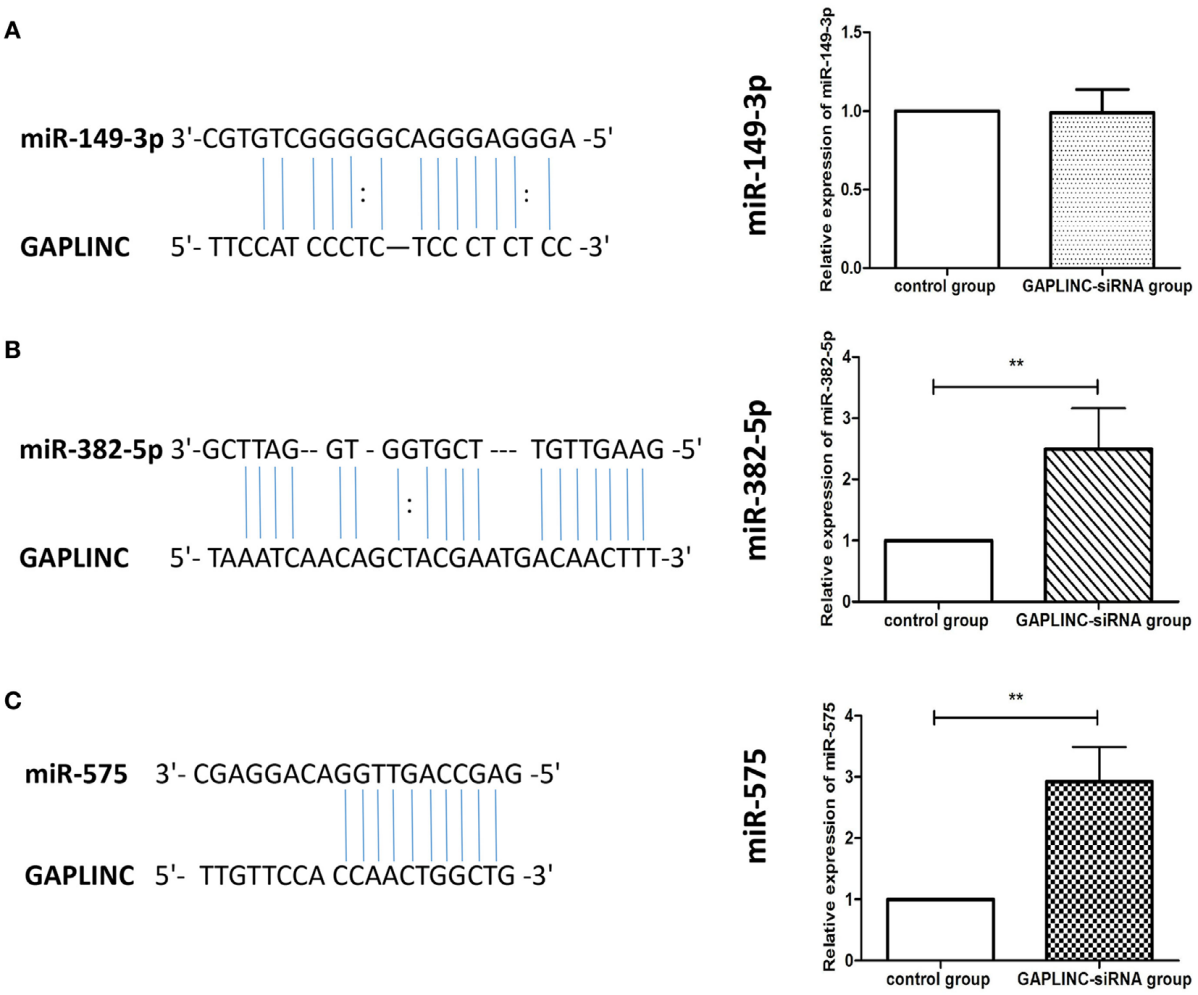
The verification of the target microRNAs of LncRNA GAPLINC (miR-149-3p, miR-382-5p, miR-575). In both **(A–C)**, the left panel shows the potential binding site in the sequences of GAPLINC with relevant microRNA, analyzed through bioinformatics; the right panel identified the relative expression of three microRNAs between negative control and GAPLINC-small interference RNA groups, respectively, measured by quantitative real-time reverse transcription PCR. **P < 0.01 vs control group.

Following a preliminary understanding of the GAPLINC-microRNA-mRNA network by the bioinformatics analysis, three candidate microRNAs (hsa-miR-575, hsa-miR-149-3p, hsa-miR-382-5p) were selected to make a binding site prediction with this methodology. Since the results of this analysis suggested that there may be a targeting relationship between GAPLINC and miR-149-3p, miR-382-5p and miR-575, the relative expression of these miRNAs between NC group and GAPLINC-siRNA groups was further measured by qRT-PCR. Although GAPLINC inhibition induced a significant upregulation of miR-382-5p and miR-575 as compared with the control group, there were no statistically significant changes in miR-149-3p detection in two groups (Figure 5). These results suggested that miR-382-5p and miR-575 may be the direct target of GAPLINC.

## Discussion

The results of the present study have particular relevance to RA, one of the most prevalent chronic inflammatory autoimmune diseases characterized by synovial hyperplasia, cartilage destruction, and joint dysfunction. Although the precise etiology and pathogenetic mechanism(s) of RA are still unknown, significant progress has been made in recent years with regard to the role of susceptibility genes, environmental insults, epigenetic modifications, and posttranslational events that initiate and promulgate the pathologic changes associated with RA (16, 17). Recent evidence has suggested that at the transcriptional or posttranscriptional level, a number of non-coding RNAs (ncRNAs) serve as master regulators that affect the expression levels of dozens or even hundreds of target genes (18). A major research effort has been directed to the study of the LncRNAs, particularly, the mRNA-like transcripts lengthier than 200 nucleotides that have no or little protein-coding capacity but play critical functional roles in a variety of biological processes, such as transcription, splicing, and translation (19). Thus, there is considerable research evidence to suggest that LncRNAs may participate in the pathophysiology of many human diseases including RA (20, 21).

In the field of oncology research, some of LncRNAs were chosen as diagnostic and prognostic biomarkers of disease susceptibility because of their superior potential as markers of tumor invasion and metastasis (22). In the forefront of RA research, LncRNAs recently have also received extensive attention as key predictive markers of disease susceptibility. Evidence implicating LncRNAs in RA is largely derived from two major lines of investigation: expression studies and genome-wide association studies. In a study by Zhang et al., the LncRNA expression profile in FLSs from patients with RA and control human subjects was compared. It was found that although 135 LncRNAs were differentially expressed to a greater degree in FLSs from patients with RA than from healthy control subjects, their role was not further confirmed (23). In another study by Song et al., not only were LncRNA signatures identified in blood mononuclear cells and serum exosome of RA patients but also notably high expression levels of LncRNA Hotair were found associated with significantly decreased levels of MMP-2 and MMP-13. This study provides empirical evidence that one LncRNA, Hotair, could be a potential biomarker for diagnosing RA (24). As sufficient data are still lacked in this important area, further studies will be required to definitively clarify the role and underlying mechanisms responsible for more critical functional LncRNAs in RA.

LncRNA GAPLINC, a newly identified functional LncRNA, also known as a positive CD44 Regulator, was recently described in patients with gastric and colorectal cancer (12–14). In these studies, GAPLINC was not only overexpressed in those tumor tissues but was also found to promote tumor cell behaviors, such as proliferation, migration, and invasive manifestations that associate with poor prognosis in patients. Hypoxia-inducible factor (HIF-1α) was found to both bind to the promoter region of GAPLINC and activate its transcription (13). Another research study provided evidence suggesting that manipulating GAPLINC expression not only altered CD44 mRNA abundance but also neutralized the effects of GAPLINC on cell migration and proliferation by suppressing CD44 expression. Mechanistically, GAPLINC was found to regulate CD44 as a molecular decoy for miR-211-3p, a microRNA that targets both CD44 and GAPLINC (12). These recent studies suggest that GAPLINC may provide a molecular attractions platform for microRNA or mRNA to play a regulatory role.

Fibroblast-like synoviocytes of RA (RA-FLS), comprise the predominant cellular population in synovium, are activated in a chronic inflammatory environment and share some of the properties of tumor cells. Some publications report RA synovial tissue to have a hypoxia microenvironment with elevated levels of HIF-1α (25, 26). As described previously, HIF-1α could activate the transcription of GAPLINC. Taken into consideration collectively, these observations raise the possibility that levels of GAPLINC may also be overexpressed in RA synovial cells that could play a regulatory role in promoting cell pathological behaviors.

In summary, the results of the present study demonstrate that the expression of GAPLINC was highly elevated in RA-FLSs compared to normal FLSs from injury patients, using qRT-PCR as the technology probe of verification. We then provided more evidence that the elevated expression of GAPLINC was functionally relevant to the tumor-like features of RA-FLSs. The finding of diminished GAPLINC expression by gene-specific interference technology was adopted in functional investigations including cell proliferation, migration, and invasion assays. Synovial swelling is a significant feature of RA, which is attributable to the inflammatory hyperplasia of synovial cells, especially the FLSs. The results of the CCK8 cell viability assay showed GAPLINC knockdown could markedly suppress the proliferation of RA-FLSs in a time-dependent fashion, suggesting that GAPLINC may be involved in the regulation of RA-FLSs growth. Bone erosion and joint damage is another major characteristic of RA. Previous studies suggested that the early inflammatory environment stimulated synovial cells to migrate and invade intra-articular structures and ultimately lead to the damage of cartilage and subchondral bone (4, 27). In animal experiments, RA-FLS was shown to have the capacity to migrate to distant joints and induce bone tissue erosion in SCID mice even without external stimulation (28). These findings suggest that the potent migratory and invasive ability of RA-FLS cannot be ignored. Our study has definitively demonstrated that GAPLINC suppression could positively depress the migration and invasive capacity of RA-FLSs, as reflected by the reduced numbers of transmembrane cells in Transwell chambers, suggesting that GAPLINC may play an important regulatory role in the migration and invasion of RA-FLSs. Additionally, RA-FLS also promote RA pathological process through secretion of a variety of proinflammatory cytokines or proteinases such as IL-6, IL-8, and MMPs (29). Multiple proinflammatory cytokines in synovial fluid stimulate a series of inflammatory reactions and MMPs contribute to cartilage and joint damage (30–32). The results found that GAPLINC inhibition reduced IL-6, IL-8, and MMP-9 production of RA-FLSs, suggesting another important function of RA-FLS affected by GAPLINC.

Until recently, most studies supported the idea that LncRNAs could modulate a wide variety of biological functions through their interactions with other biomolecules: e.g., DNA, RNA, and protein. The LncRNAs and their interactions, therefore, play diverse functional roles through key pathways. Among them, LncRNA–microRNA duplexes were the most frequently reported (33). Recently, an alternative proposal of ceRNA hypothesis was put forward suggesting that microRNA could mediate cross-talks between LncRNAs and mRNAs and that LncRNAs may perform their functions as a molecular sponge of microRNAs (34, 35).

**Figure 6:**
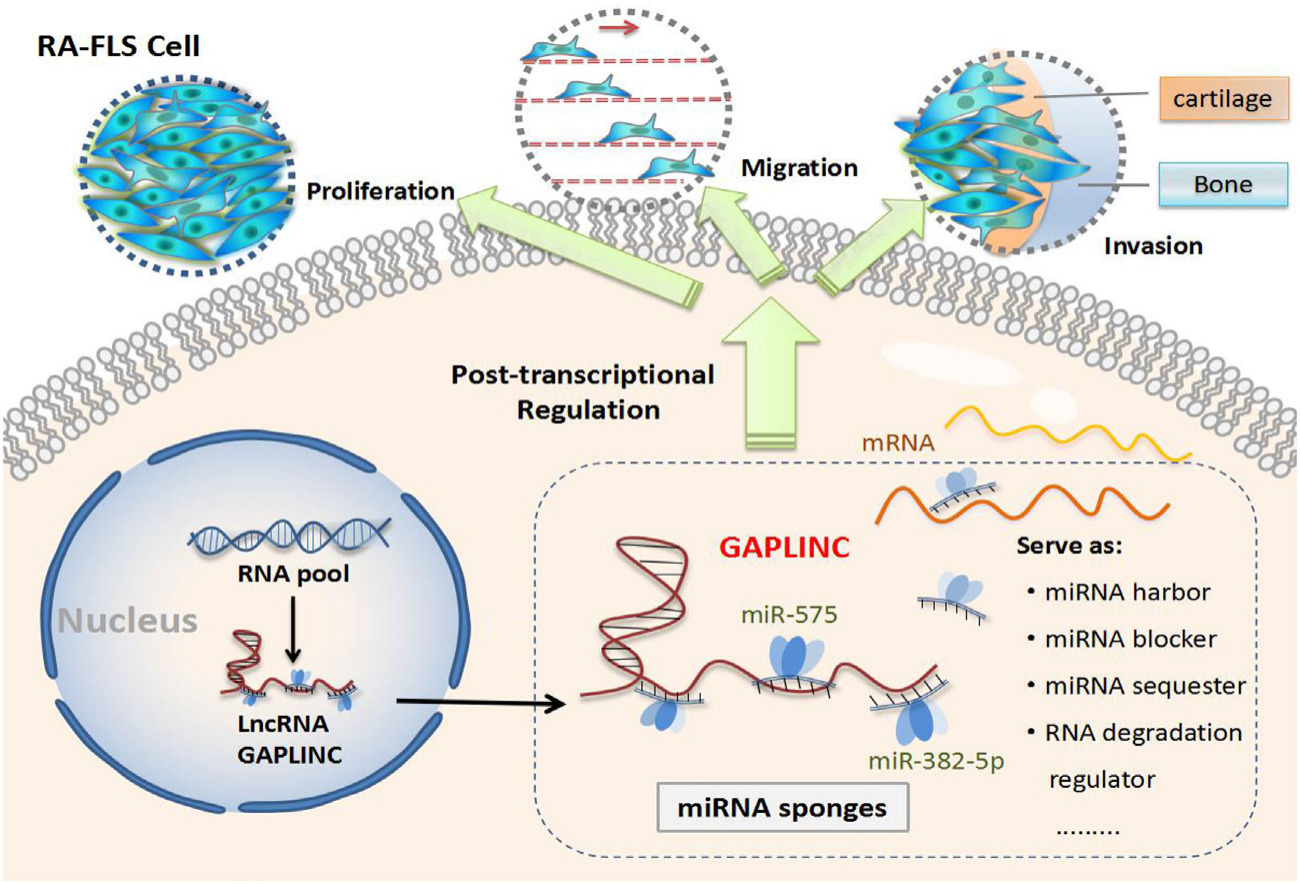
The schematic representation showing possible mechanism of LncRNA GAPLINC. The high expression level of GAPLINC may regular cell behaviors in rheumatoid arthritis (RA)-fibroblast-like synoviocytes (FLSs) though a post-transcriptional way. Bioinformatics analysis speculated that GAPLINC may be molecular attractions for microRNAs and have some regulatory roles. In this investigation, the expression of miR-382-5p and miR-575 had significant increases after GAPLINC suppression, suggesting GAPLINC may promote RA-FLS tumor-like behaviors in an miR-382-5p-dependent and miR-575-dependent manner.

In exploring the potential mechanism of GAPLINC, we performed a bioinformatics analysis of GAPLINC to explore its interactions and targets. The related microRNAs of GAPLINC were predicted using miRanda, PITA, RNAhybrid algorithms. The minimum free energy, seed region, and microRNA recognition elements of LncRNA–microRNA duplexes were calculated to choose superior candidates (36). We found 64 microRNAs may be the interactions of GAPLINC, including hsa-miR-575, hsa-miR-149-3p, hsa-miR-382-5p, hsa-miR-516a-3p, hsa-miR-1184, hsa-miR-1261, hsa-miR-3127-5p, hsa-miR-4649-3p, and so on. Some of them have been already reported in oncology. The related pathway and downstream genes of GAPLINC were predicted using TargetScan, miRDB and miRanda softwares, KEGG database, and GO analysis. We selected six microRNAs as superior candidates (hsa-miR-575, hsa-miR-149-3p, hsa-miR-382-5p, hsa-miR-516a-3p, hsa-miR-1184, hsa-miR-1261) according to their comprehensive assessments and constructed a co-action network graph. As a result, we have obtained a better understanding of the possible functioning mode of GAPLINC. Next, we selected top three candidate microRNAs (hsa-miR-575, hsa-miR-149-3p, hsa-miR-382-5p) to construct a binding site prediction. The results suggest that there may be a targeting relationship between GAPLINC and miR-149-3p, miR-382-5p, or miR-575. We further verified the expression of three microRNAs (hsa-miR-575, hsa-miR-149-3p, hsa-miR-382-5p) between NC and GAPLINC-siRNA groups using qRT-PCR. The results, thus far, show GAPLINC induced suppression to be a significant upregulation mechanism of miR-382-5p and miR-575 as compared with control group. Since there were no statistically significant changes in miR-149-3p detection in two groups, it is likely that miR-382-5p and miR-575 may be the direct targets of GAPLINC. A research group found that microRNA-382-5p aggravates breast cancer progression by regulating the RERG/Ras/ERK signaling axis. In this study, miR-382-5p was found to promoted breast cancer cell viability, clonogenicity, survival, migration, invasion, thereby, it was thought to be a oncomiR for the breast cancer cell (37). Another study was designed to reveal the function of miR-575 in non-small cell lung cancer (NSCLC). Researchers found miR-575 might function as an oncogene by directly targeting BLID to regulate the proliferation, migration, and invasion of NSCLC cells (38). Based on these observations, miR-382-5p and miR-575 could be highly related to cell pathologic behaviors. Recent researches also found that LncRNAs display a regulatory role at several molecular points of the post-transcriptional level, e.g., miRNA harbor, miRNA sequester, miRNA blocker, RNA degradation regulator, RNA splicing regulator, RNA editing regulator, translational efficiency regulator (39). We suggest that GAPLINC may be a molecular decoy for microRNAs, which function to harbor the recognition site for functional microRNAs, displace the microRNAs from their mRNA targets or directly compete with microRNAs for binding the same mRNAs (Figure 6).

Collectively, our studies strongly suggest that elevated GAPLINC expression promote the tumor-like biologic features of RA-FLSs. Targeting GAPLINC could significantly impair the ability of RA-FLSs cell proliferation, migration, and invasion. We made a preliminary bioinformatics analysis of GAPLINC gene sequence, and constructed the GAPLINC-microRNA-mRNA network using advanced databases. The GAPLINC may though its molecular interactions to play diverse biologic roles. The development of molecular targeted drugs is the trend of the future treatment (40). Our investigation of GAPLINC, a novel molecule, may provide some new insight for a novel therapeutic approach of RA patients.

## Ethics Statement

All synovial specimens were obtained from human discarded tissues. The research was approved by the ethics committee for clinical medical research at Third Affiliated Hospital of Sun Yat-sen University (No.[2017]2-169) and conducted after receiving informed consent from study subjects.

## Author Contributions

SZ, YP, and BM conception and design, data analysis and interpretation, manuscript writing, final approval of manuscript. BM, XG, MY, FL, YL, XB, and JW performed experiments and data collection. LF and XL collected synovial tissues. JB edited manuscript.

## Conflict of Interest Statement

The authors declare that the research was conducted in the absence of any commercial or financial relationships that could be construed as a potential conflict of interest.
