# Supplementary material for: Long Non-Coding RNA GAPLINC Promotes Tumor-Like Biologic Behaviors of Fibroblast-Like Synoviocytes as MicroRNA Sponging in Rheumatoid Arthritis Patients
Source: Front Immunol. 2018 Apr 10;9:702. doi: 10.3389/fimmu.2018.00702 (PMC5902673; doi:10.3389/fimmu.2018.00702)
Supplement: Supplementary file 1 [file Image_1.pdf]

Supplementary material

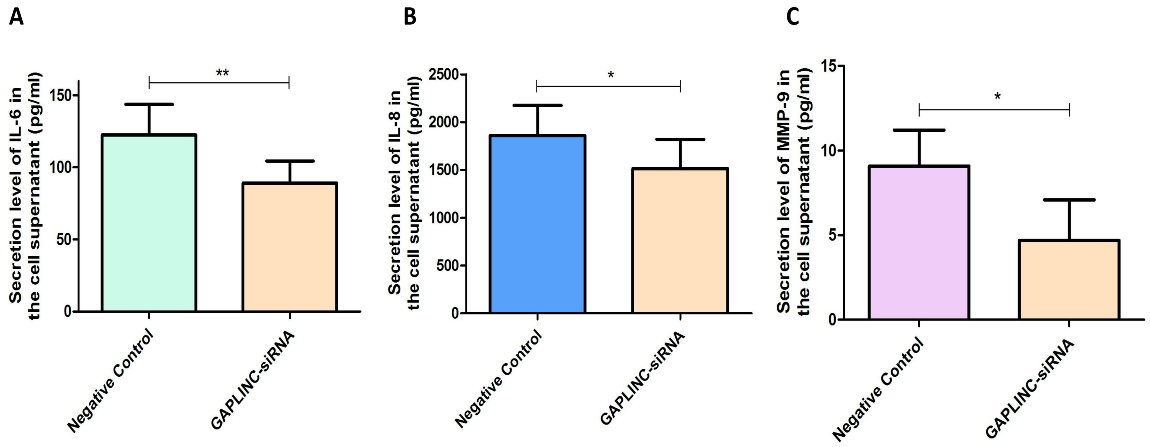

Supplementary figure 1 GAPLINC suppression decreases IL-6, IL-8 and MMP-9 production of RA-FLSs. The concentrations of proinflammatory cytokines (IL-6, IL-8) and matrix metalloproteinases-9 (MMP-9) (pg/mL) in the supernatant of RA-FLS cells were reduced after GAPLINC-siRNA treatment, compared to the NC-siRNA group (*P<0.05*).
