# Supplementary material for: Long Non-Coding RNA GAPLINC Promotes Tumor-Like Biologic Behaviors of Fibroblast-Like Synoviocytes as MicroRNA Sponging in Rheumatoid Arthritis Patients
Source: Front Immunol. 2018 Apr 10;9:702. doi: 10.3389/fimmu.2018.00702 (PMC5902673; doi:10.3389/fimmu.2018.00702)
Supplement: Supplementary file 2 [file Table_1.docx]

**Supplementary table 1 Sequence of primer sets and specific siRNA sequences used in this study**

| **Oligonucleotides** | **Sequences** |
| --- | --- |
| **GAPDH-primer** | **F: 5’-AAGGTGAAGGTCGGAGTCAAC-3’**  **R: 5’-GGGGTCATTGATGGCAACAATA-3’** |
| **LncRNA GAPLINC- primer** | **F: 5’-ACACACAGCAGCCTGGTTTC-3’**  **R: 5’-ATGGCACAATCAGGGCTCTT-3’** |
| **N.C - siRNA** | **F: 5-UUCUCCGAACGUGUCACGUTT-3′ R: 5-ACGUGACACGUUCGGAGAATT-3′** |
| **GAPLINC-siRNA-461** | **F: 5-GCACUUAGAAGAACCAAUATT-3′ R: 5-UAUUGGUUCUUCUAAGUGCTT-3′** |
